# Reference values of myocardial native T1 and extracellular volume in patients without structural heart disease and had negative 3T cardiac magnetic resonance adenosine stress test

**DOI:** 10.1016/j.ijcha.2023.101181

**Published:** 2023-01-30

**Authors:** Weerapat Kositanurit, Nonthikorn Theerasuwipakorn, Yongkasem Vorasettakarnkij, Kanokvalee Ponkanist, Chonthicha Lerdkhonsan, Monravee Tumkosit, David C. Wendell, Pairoj Chattranukulchai

**Affiliations:** a Department of Physiology, Faculty of Medicine, Chulalongkorn University, King Chulalongkorn Memorial Hospital, Bangkok 10330, Thailand; b Division of Cardiovascular Medicine, Faculty of Medicine, Chulalongkorn University, Cardiac Center, King Chulalongkorn Memorial Hospital, Bangkok 10330, Thailand; c Division of Hospital and Ambulatory Medicine, Faculty of Medicine, Chulalongkorn University, King Chulalongkorn Memorial Hospital, Bangkok 10330, Thailand; d Department of Radiology, Faculty of Medicine, Chulalongkorn University, King Chulalongkorn Memorial Hospital, Bangkok 10330, Thailand; e Duke Cardiovascular Magnetic Resonance Center, Division of Cardiology, Duke University Medical Center, Durham, NC 27708, USA

**Keywords:** Native T1 mapping, Extracellular volume, 3 Tesla, Cardiac magnetic resonance

## Abstract

**Background:**

To establish the reference values of native T1 and extracellular volume (ECV) in patients without structural heart disease and had a negative adenosine stress 3T cardiac magnetic resonance.

**Methods:**

Short-axis T1 mapping images were acquired using a modified Look-Locker inversion recovery technique before and after administration of 0.15 mmol/kg gadobutrol to calculate both native T1 and ECV. To compare the agreement between measurement strategies, regions of interest (ROI) were drawn in all 16 segments then averaged to represent mean global native T1. Additionally, an ROI was drawn in the mid-ventricular septum on the same image to represent the mid-ventricular septal native T1.

**Results:**

Fifty-one patients (mean 65 years, 65 % women) were included. Mean global native T1 averaged from all 16 segments and a mid-ventricular septal native T1 were not significantly different (1221.2 ± 35.2 vs 1228.4 ± 43.7 ms, p = 0.21). Men had lower mean global native T1 (1195 ± 29.8 vs 1235.5 ± 29.4 ms, p < 0.001) than women. Both mean global and mid-ventricular septal native T1 were not correlated with age (r = 0.21, p = 0.13 and r = 0.18, p = 0.19, respectively). The calculated ECV was 26.6 ± 2.7 %, which was not influenced by either gender or age.

**Conclusions:**

We report the first study to validate the native T1 and ECV reference ranges, factors influencing T1, and the validation across measurement methods in older Asian patients without structural heart disease and had a negative adenosine stress test. These references allow for better detection of abnormal myocardial tissue characteristics in clinical practice.

## 1 Introduction

Longitudinal relaxation time (T1) is a time constant representing the recovery of longitudinal magnetization after applying a radiofrequency pulse. [1], [2] Several myocardial abnormalities such as myocardial edema [3], [4], fibrosis [5], or cardiac amyloid deposition [6] have been shown to alter the natural T1 of myocardium. Previously, these changes in T1 were detected using T1-weighted sequences, which show abnormal regions as bright compared to normal myocardium. Recently, cardiac magnetic resonance (CMR) imaging groups have developed methods to actually calculate the T1 of different tissues, referred to generally as T1 mapping. [3], [7], [8], [9] Hence the abnormalities seen in these disease states may be detected more directly by using these new T1 mapping techniques. Pre-contrast or native T1 is the measurement of T1 time prior to administration gadolinium-based contrast agents (GBCA), which shortens T1 value. In clinical practice, native T1 is used as an alternative or adjunctive to late gadolinium enhancement (LGE) imaging for the diagnosis of myocardial diseases.[8], [10], [11].

Extracellular volume (ECV) uses T1 values obtained pre- and post-contrast to calculate the extracellular component of the myocardium. The formula for estimation of ECV is derived from myocardial and blood T1 values before and after GBCA administration as well as the patient’s hematocrit. [9] An increase of ECV represents extracellular compartment expansion due to water, amyloid, collagen deposition, or other infiltrative processes. [7], [9] Recently, native T1 mapping and ECV assessment have emerged as methods for characterizing changes in various cardiac conditions in a clinical scan. However, the normal reference values of native T1 are varied depending upon the magnetic field strength, magnetic field inhomogeneities, acquisition schemes, and patient characteristics. [12] Most previous research was studied in relatively young, healthy volunteers. [13], [14], [15] The data for low-to-intermediate cardiovascular risk older patients with normal structural heart, which are majority of subjects referred for clinical scan, is scarce. Thus, we decide to validate the normal reference values and associated factors for native T1 and ECV in patients without structural heart disease and no ischemia as defined by a negative CMR adenosine stress test.

## 2 Materials and methods

### 2.1 Study population

Adult patients with low-to-intermediate risk of cardiovascular disease (CVD) referred for clinical stress CMR scan due to suspected coronary artery disease were prospectively recruited at King Chulalongkorn Memorial Hospital, during July 2020 to January 2021. The study protocol was approved by the committees of Institutional Review Board, Faculty of Medicine, Chulalongkorn University. The need for consent was waived by an IRB (No. 302/64). All procedures performed in studies involving human participants were in accordance with the World Medical Association’s Declaration of Helsinki.

The exclusion criteria were 1) patients under 18 years old, 2) any evidence of CVD as indicated by clinical history and physical exam, 3) history of diabetic mellitus, 4) pregnancy, 5) abnormal electrocardiographic (ECG) findings or presence of both atrial and ventricular arrhythmia at scan date, 6) abnormal structural heart conditions including abnormal ventricular function, chamber size, myocardial wall thickness and mass, moderate-to-severe valvular dysfunction or the presence of a scar on delayed enhancement imaging, and 7) artifact on pre-contrast T1 imaging, which compromise the accuracy of measuring the native T1 value. Baseline demographic data such as comorbidity and anthropometric parameters were collected as well as essential laboratory results including serum creatinine and hematocrit on the day of the CMR scan.

### 2.2 CMR acquisition

The scan was performed using 3 Tesla (Magnetom Vida; Siemens Healthineers, Erlangen, Germany) using an18-channel cardiac phased array receiver. The stress perfusion protocol included cines, pre-contrast T1 mapping, first pass perfusion, late gadolinium enhancement (LGE), and post-contrast T1 mapping. To assess left ventricular (LV) function and mass, two-, three-, and four-chamber long-axis images and 12 consecutive 8 mm short-axis cine images were acquired using a steady-state free precession (SSFP) sequence. Then, three short-axis images at basal, mid, and apical LV were acquired for pre-contrast T1 mapping using Modified look locker (3(3)3(3)5 MOLLI) technique. [16] The measurement parameters for T1 map are described in **Supplementary table 1**. The CMR stress test was performed by administering adenosine (140 µg/kg/min for total 3.5 min). Once adequate vasodilatory effect was achieved as confirmed by an increase in heart rate or patient attestation, a 0.10 mmol/kg of gadobutrol (Gadovist, Bayer Healthcare, Leverkusen, Germany) was administered. During the gadobutrol infusion a first-pass perfusion sequence was run, which obtains base, mid, and apical single-shot fast gradient echo (GRE) images during each heartbeat. Seven minutes after the 1st gadobutrol injection, an additional 0.05 mmol/kg of gadobutrol was added then a 2D segmented inversion recovery (GRE) sequences were obtained to detect myocardial scar. Lastly, post-contrast T1 mapping images were acquired in identical slice locations as pre-contrast T1 mapping. Post-contrast T1 maps were acquired approximately 15 min after the 1st gadobutrol injection to approach steady-state conditions. [12].

### 2.3 Post-processing CMR analysis

**Fig. 1:**
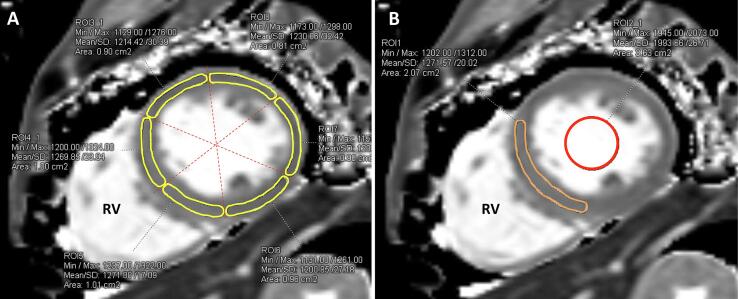
The methods for drawing region of interest (ROI) on grey scale images of native T1 map. A) ROIs were drawn along each of 16 segments of the left ventricle to obtain per-segment native T1. The average T1 value from all segments represents the mean global native T1. B) A single ROI in mid-ventricular septum was drawn on the same image to represent the mid-ventricular septal native T1. Another ROI was placed in the center of the blood pool at the mid-ventricular short-axis view for calculating extracellular volume. RV; right ventricle.

CMR images were checked to verify a diagnostic image quality. Two experienced observers blinded to clinical data analyzed the images. The image interpretation and post-processing were performed with post-processing software (*syngo*.via, Siemens Healthineers, Erlangen, Germany), including LV chamber and function quantification, pre- and post-contrast T1 mappings, and ECV. For LV chamber assessment, the endocardial and epicardial contours were automatically delineated in diastole and systole in a stack of short-axis slices of the whole LV excluding papillary muscles and trabeculae, with manual adjustments when needed. The calculated parameters included LV end-diastolic volume, end-diastolic volume indexed to body size, ejection fraction, and mass index. Post-processing of native T1 mapping involved manually drawing regions of interest (ROIs) on grey scale short-axis images of T1 map in each of 16 segments of LV myocardium in accordance with standardized myocardial segmentation of the American Heart Association to obtain per-segment native T1 (Fig. 1**A**). The T1 values from all segments were averaged together to calculate a mean global native T1. In addition, we drew a single ROI in the mid-ventricular septum representing the mid-ventricular septal native T1 in order to compare agreement between measurement methods (Fig. 1**B**). We used the single ROI in the mid-ventricular septum and ROI placed in the center of blood pool on pre- and post- contrast T1 maps to calculate ECV using the formula ECV (%) = (100-Hct) * [Δ(1/myocardial T1) ∕ Δ(1/blood T1)].[12].

### 2.4 Statistical analysis

Statistical analysis was performed using SPSS (version 22.0, IBM Inc., Chicago, IL). Continuous data were presented in mean ± standard deviation (SD). Categorical data were reported as frequencies and percentages. Ninety-five percent confident interval (95 % CI) of native T1 was used to define the normal value of native T1. To determine the difference between independent groups, an independent *t*-test or Chi-square was used. The relationship of native T1 value and ECV with age were compared using Pearson’s correlation coefficient. Bland-Altman plot and intra-class correlation coefficient (ICC) were used to compare two different methods of native T1 measurement. Two experienced cardiologists independently tested inter-observer reproducibility. Statistical significance was indicated as p-value of less than 0.05.

## 3 Results

Of 335 patients scheduled for CMR adenosine stress testing at 3T between July 2020 and January 2021, 204 patients had a negative stress result. Ninety-two patients were excluded due to presence of diabetes or known CVD, and 61 patients were excluded due to presence of abnormal ECG or structural heart conditions. The final population consisted of 51 patients. The majority of patients (92 %) were referred due to atypical chest discomfort with or without worsening dyspnea. The mean age was 65 ± 12 years, 65 % woman. The majority of patients were from age groups 61 to 70 (16, 31.4 %) followed by 51 to 60 (14, 27.5 %), and 71 to 80 (13, 25.5 %). The baseline characteristics of patients according to gender are shown in Table 1. Briefly, men had higher body surface area (1.8 ± 0.2 vs 1.6 ± 0.2 m^2^, p = 0.001), lower resting systolic blood pressure (122.1 ± 23.6 vs 141.9 ± 23.6 mmHg, p = 0.006), and higher hematocrit (43.1 ± 4.3 vs 38.9 ± 1.8 %, p < 0.001) than women.

**Table 1:** Baseline characteristics according to gender.

	**All (n = 51)**	**Female (n = 33)**	**Male (n = 18)**	**p-value**
Age (years)	65 ± 12	67 ± 11	61 ± 12	0.07
BMI (kg/m^2^)	25.6 ± 5.2	25.8 ± 4.9	25.3 ± 5.9	0.77
BSA (m^2^)	1.7 ± 0.2	1.6 ± 0.2	1.8 ± 0.2	0.001*
Hypertension	20 (39)	14 (42)	6 (33)	0.12
Dyslipidemia	28 (55)	19 (58)	9 (50)	0.34
Smoking	1 (2)	0	1	0.86
Resting HR (bpm)	68 ± 11.5	69 ± 11.8	66 ± 8.3	0.36
Resting SBP (mmHg)	134.9 ± 25.3	141.9 ± 23.6	122.1 ± 23.6	0.006*
GFR (ml/min/1.73 m^2^)	83 ± 20	81 ± 19	87 ± 21	0.35
Hematocrit (%)	40.2 ± 3.4	38.9 ± 1.8	43.1 ± 4.3	< 0.001*

Data are presented as mean (SD) or number (percent).

BMI: body mass index; BSA: body surface area; HR: heart rate; SBP: systolic blood pressure; GFR: glomerular filtration rate.

* Indicate statistical significance (p < 0.05).

**Fig. 2:**
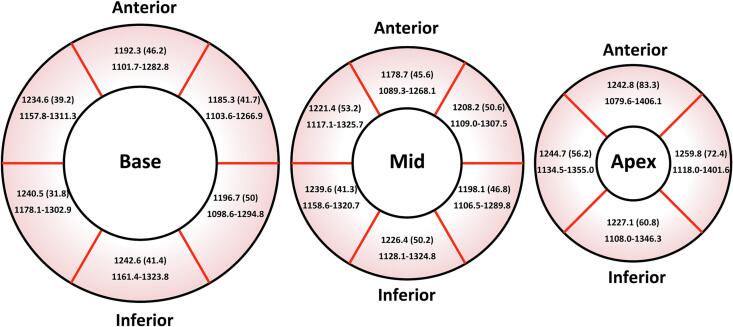
The mean (SD) and 95% CI of per-segment native T1 values (ms) from the basal-to-apical short-axis view of the left ventricular in all patients.

Table 2 demonstrates the CMR parameters. LV ejection fraction was higher in women than in men (66 ± 5.3 vs 60 ± 4.6 %, p < 0.001). The LV end-diastolic volume index and LV mass index were higher in men than in women (72.3 ± 13.3 vs 65.5 ± 10.1, p = 0.047 and 63.2 ± 9.3 vs 54.2 ± 9.1 g/m^2^, p = 0.001, respectively). Overall, there was no significant difference between mean global and mid-ventricular septal native T1 values (1221.2 ± 35.2 vs 1228.4 ± 43.7 ms, p = 0.21). The Bland-Altman plot also revealed good agreement between these measurement methods with 96 % of ICC (Supplementary Fig. 1). Men had lower mean global native T1 (1195 ± 29.8 vs 1235.5 ± 29.4 ms, p < 0.001) and mid-ventricular septal native T1 (1209.9 ± 46.4 vs 1238.5 ± 39.2 ms, p = 0.02) than women. Both global and mid-ventricular septal native T1 were not correlated with age (r = 0.21, p = 0.13 and r = 0.18, p = 0.19, respectively). However, patients younger than 65 years old had significantly lower global native T1 (1207.5 ± 23.9 vs 1234.3 ± 39.5 ms, p = 0.005) and lower mid-ventricular septal native T1 (1215.7 ± 45.6 vs 1240.1 ± 38.7 ms, p = 0.04) when compared with those aged 65 years or above. The reference range of native T1 values in each 16 segments from the basal-to-apical short-axis view of the LV in all patients are shown in Fig. 2.

**Table 2:** CMR parameters according to gender.

	**Total patient (n = 51)**	**Female (n = 33)**	**Male (n = 18)**	**p-value**
LVEF (%)	64 ± 5.8	66 ± 5.3	60 ± 4.6	< 0.001*
LVEDV (ml)	115 ± 28.5	105.5 ± 20	132.5 ± 33.7	0.001*
LVEDVi (ml/m^2^)	68 ± 11.7	65.5 ± 10.1	72.3 ± 13.3	0.047*
LVMI (g/m^2^)	57.4 ± 10	54.2 ± 9.1	63.2 ± 9.3	0.001*
Mean global native T1 (ms)	1221.2 ± 35.2	1235.5 ± 29.4	1195 ± 29.8	< 0.001*
Mid-ventricular septal native T1 (ms)	1228.4 ± 43.7	1238.5 ± 39.2	1209.9 ± 46.4	0.02*
ECV (%)	26.6 ± 2.7	27 ± 1.8	25.7 ± 3.9	0.17

Data are presented as mean (SD).

ECV: extracellular volume; LVEF: left ventricular ejection fraction; LVEDV: left ventricular end-diastolic volume; LVEDVi: left ventricular end-diastolic volume index; LVMI: left ventricular mass index.

* Indicate statistical significance (p < 0.05).

For the ECV analysis, the overall value was 26.6 ± 2.7 % without statistically significant difference between genders (25.7 ± 3.9 % for male and 27 ± 1.8 % for female, p = 0.17). There was no significant correlation between ECV and age (r = 0.28, p = 0.07).

The inter-observer reproducibility was 93 % for the per-segment native T1 measurement and 95 % for the mid-ventricular septal native T1 measurements. Mean global native T1 averaging from the value of all 16 segments provided the inter-observer reliability of 99 %. The intra-observer reproducibility was 99 % for the per-segment native T1 measurement, 95 % for the mid-ventricular septal native T1 measurements, and 99 % for the mean global native T1.

## 4 Discussion

This is the first study to validate the native T1 and ECV reference ranges, factors influencing T1, and the validation across measurement methods in older Asian patients without structural heart disease and had a negative 3T CMR adenosine stress test.

While several previous studies reported the normal reference of T1 and ECV in healthy subjects, a population-based reference values remain necessary. [1] Since it is varied depending upon the type of pulse sequence, acquisition schemes, and magnetic field inhomogeneity. In addition, different patient characteristics also contribute to such a variation. [12], [17] The mean of the reference value of native T1 acquired from 3T CMR with MOLLI technique in normal subjects was approximately 1050–1240 ms in the literature. [14], [15], [18], [19], [20], [21] The reference value from our study is at the higher end of the range (mean global and mid-ventricular septal native T1 values were 1221.2 ± 35.2 and 1228.4 ± 43.7 ms, respectively) compared to prior reports. It is possible that this results from the inter‐vendor T1 variability and different characteristics of the subjects. We studied low-to-intermediate CVD risk patients whose 39 % of them have controlled hypertension with normal structural heart including LV mass. The chronic elevation of blood pressure could affect the extracellular change results in an increase of native T1, which occurs prior to the change of LV mass index. [9].

In the literature the age dependence for normal T1 value has been conflicting to date. Roy et.al demonstrated the positive correlation between age and native T1 value. [15] However, Liu et.al reported no significant influence of age for T1 values at 3T in an African-American population. [21] The majority of patients in our study were older adults (mean age 65 years) while the mean age of subjects in most previous reports was under 55 years. [14], [18], [19], [20] This might be one of the reasons that native T1 value in our study was slightly higher than previous literatures. From our results, although there was no significant correlation between age and native T1 overall, native T1 value was significantly higher in patients older than 65 years of age.

Gender is another factor affecting native T1 value. [13], [15], [20], [22] In line with previous studies, we confirmed that native T1 was significantly greater in females than in males. Conversely, ECV was less dependent on age or gender. Our results were consistent with the majority of the studies that revealed the normal value of ECV about 25–27 % without age and gender difference. [14], [15], [20].

There are multiple methods to measure native T1 from T1 mapping images. ROIs in abnormal areas on visual inspection should be drawn in focal myocardial disease. [1], [12] In the absence of focal disease, there are two commonly used ROI methods when analyzing native T1 in diffuse disease. Drawing a single ROI at the mid-ventricular septum has been preferred to placing ROIs along each of 16 segments of LV to represent the global assessment in diffuse disease. [1], [12] We performed both methods in post-processing analysis and found that both provided similar performance with good robustness as the previous study. [15] Hence, drawing a single ROI at the mid-ventricular septum to measure the native T1 value of diffuse myocardial disease may be sufficient, less time-consuming compared to a 16-segment analysis in a relatively healthy patient.

The study has some limitations. Firstly, this is a single-center and vendor-specific study; hence, this may limit its generalizability. Secondly, the results may not represent the true reference values in a “truly” healthy subject since a GBCA administration in asymptomatic healthy subjects is a major ethical concern. Instead, we recruited low-to-intermediate CVD risk patients referred for clinical scanning who had normal structural heart, including a negative adenosine stress CMR. Consequently, we decided to validate the normal references in this population, which are a majority of subjects come to the scanner in real-life practice. Lastly, ECV values were calculated after a 0.15 mmol/kg of gadobutrol injection, slightly different ECV values have been reported when different doses of contrast media were used. [15].

Despite the aforementioned limitations, this study shows the robustness of native T1 measurement demonstrated by high inter-and intra-observer reproducibility. This provides confidence in results when serially following patients with these sequences.

## 5 Conclusions

We provide normal native T1 and ECV reference ranges, factors influencing native T1, and the validation across measurement methods in patients without structural heart disease and who had a negative 3T CMR adenosine stress test. Females have higher native T1 than males without difference in ECV. These reference values allow for better detection of abnormal myocardial tissue characteristics in clinical practice.

## Declaration of Competing Interest

The authors declare that they have no known competing financial interests or personal relationships that could have appeared to influence the work reported in this paper.
