# Supplementary material for: Reference values of myocardial native T1 and extracellular volume in patients without structural heart disease and had negative 3T cardiac magnetic resonance adenosine stress test
Source: Int J Cardiol Heart Vasc. 2023 Jan 30;45:101181. doi: 10.1016/j.ijcha.2023.101181 (PMC9923153; doi:10.1016/j.ijcha.2023.101181)
Supplement: Supplementary data 1 [file mmc1.docx]

**Supplementary file**

**1) Supplementary table 1.** Imaging parameter of pre- and post-contrast T1 maps

**2) Supplementary figure 1.** Bland-Altman plot of different measurement methods (mean global and mid-ventricular septal native T1)

**3) Supplementary figure 2.** Bland-Altman plots for inter-observer variability of mid-ventricular native T1

**Supplementary table 1.** Imaging parameter of pre- and post-contrast T1 maps

|  | **Native T1 Map**  **(RR interval > 700 ms)** | **Native T1 Map**  **(RR interval < 700 ms)** | **Post-contrast T1 Map**  **(RR interval > 700 ms)** | **Post-contrast T1 Map**  **(RR interval < 700 ms)** |
| --- | --- | --- | --- | --- |
| **Pulse sequence** | Single-shot TrueFISP | Single-shot TrueFISP | Single-shot TrueFISP | Single-shot TrueFISP |
| **Slice thickness (mm)** | 8 | 8 | 8 | 8 |
| **FOV (mm)** | 306.6x360 | 307.5x360 | 306.6x360 | 307.5x360 |
| **Acquisition matrix** | 144x256 | 132x192 | 144x256 | 132x192 |
| **Voxel size (mm^3^)** | 1.4x1.4x8.0 | 1.9x1.9x8.0 | 1.4x1.4x8.0 | 1.9x1.9x8.0 |
| **TR (ms)** | 280.6 | 263.9 | 360.6 | 341.4 |
| **TE (ms)** | 1.12 | 1.01 | 1.12 | 1.01 |
| **TI start (ms)** | 100 | 100 | 100 | 100 |
| **TI increment (ms)** | 80 | 80 | 80 | 80 |
| **Flip angle (degree)** | 35 | 35 | 35 | 35 |
| **Bandwidth (Hz/Px)** | 1085 | 1085 | 1085 | 1085 |

FISP: fast imaging with steady-state free precession; FOV: field of view; TE: the echo time; TR: the repetition time

**Supplementary figure 1.** Bland-Altman plot of different measurement methods (mean global and mid-ventricular septal native T1)


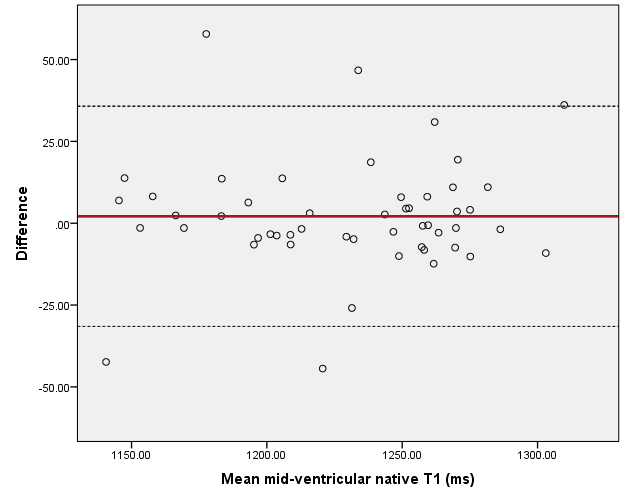


Y axis is the difference between 2 measurement methods. X axis is the mean of 2 methods. Red line represents mean difference and dot lines show ±1.96 standard deviation from mean difference. Clear circles demonstrate the difference between measurement methods in each patient.

**Supplementary figure 2.** Bland-Altman plots for inter-observer variability of mid-ventricular native T1


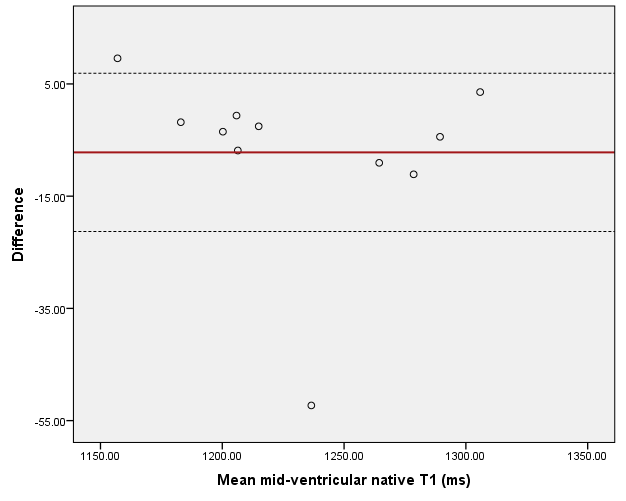


Y axis is the difference between 2 independent assessors. X axis is the mean of mid-ventricular native T1 from 2 independent assessors. Red line represents mean difference and dot lines show ±1.96 standard deviation from mean difference. Clear circles demonstrate the difference between mid-ventricular native T1 from 2 independent assessors.
